# Thermodynamic Stability of Psychrophilic and Mesophilic Pheromones of the Protozoan Ciliate *Euplotes*

**DOI:** 10.3390/biology2010142

**Published:** 2013-01-14

**Authors:** Michael Geralt, Claudio Alimenti, Adriana Vallesi, Pierangelo Luporini, Kurt Wüthrich

**Affiliations:** 1 Department of Molecular Biology, The Scripps Research Institute, La Jolla, CA 92037, USA; E-Mail: mgeralt@scripps.edu; 2 Department of Environmental and Natural Sciences, University of Camerino, Camerino 62032, Italy; E-Mails: claudio.alimenti@unicam.it (C.A.); adriana.vallesi@unicam.it (A.V.); 3 Skaggs Institute for Chemical Biology, The Scripps Research Institute, La Jolla, CA 92037, USA

**Keywords:** protein denaturation, protein stability, circular dichroism spectroscopy, psychrophilic proteins, chemical signals

## Abstract

Three psychrophilic protein pheromones (E*n*-1, E*n*-2 and E*n*-6) from the polar ciliate, *Euplotes nobilii*, and six mesophilic pheromones (E*r*-1, E*r*-2, E*r*-10, E*r*-11, E*r*-22 and E*r*-23) from the temperate-water sister species, *Euplotes raikovi*,were studied in aqueous solution for their thermal unfolding and refolding based on the temperature dependence of their circular dichroism (CD) spectra. The three psychrophilic proteins showed thermal unfolding with mid points in the temperature range 55–70 °C. In contrast, no unfolding was observed for any of the six mesophilic proteins and their regular secondary structures were maintained up to 95 °C. Possible causes of these differences are discussed based on comparisons of the NMR structures of the nine proteins.

## 1. Introduction

Protozoan ciliates represent a major micro-eukaryotic component of the polar ecosystem [1,2], which can readily be collected from every aquatic habitat for use in stable laboratory cultures [3]. Strains of *Euplotes* species such as *E. patella*, *E. raikovi*, *E. octocarinatus* and *E. crassus* inhabiting non-polar temperate waters, and of *E. nobilii* inhabiting Arctic and Antarctic waters are capable of secreting cell type-specific signaling proteins genetically specified at a single multi-allelic locus (designated as *mating-type*, or *mat* locus) [4,5]. These water-borne “pheromones” are functionally associated with the genetic mechanism of the mating types and act as prototypic autocrine (autologous) growth factors and as paracrine (heterologous) inducers of mating pair formation [6,7]. In addition to the full-length coding gene sequences [8,9,10], the three-dimensional molecular structures of a significant number of pheromones were determined by NMR spectroscopy in solution, firstly, from the temperate-water species, *E. raikovi* [11,12,13,14,15,16,17], and subsequently from the polar-water species, *E. nobilii* [18,19,20]. These mesophilic (*E. raikovi*) and psychrophilic (*E. nobilii*) pheromone families, both characterized by small, helical and disulfide-rich proteins of 37 to 63 amino acids, thus represent an interesting source of material for structure based comparative studies of protein adaptation to cold.

**Figure 1:**
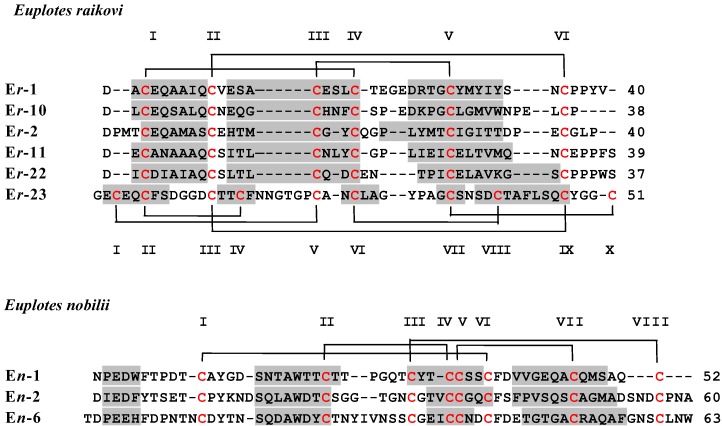
Amino acid sequences of *E. nobilii* and *E. raikovi* pheromones. The sequence alignment was maximized by insertion of gaps. Cysteines are marked in red and by progressive Roman numerals, and their pairing into disulfide bonds is indicated by brackets. The sequence regions involved in the formation of helical structures are shadowed. The PDB codes of the pheromone NMR and crystal (E*r*-1) structures are the following: E*r*-1, 1ERC, 1ERl; E*r*-2, 1ERD; E*r*-10, 1ERP; E*r*-11, 1ERY; E*r*-22, 1HD6; E*r*-23, 1HA8; E*n*-1, 2NSV; E*n*-2, 2NSW; E*n*-6, 2JMS.

Here we present data on the thermal denaturation of the three pheromones E*n*-1, E*n*-2 and E*n*-6 from the psychrophilic pheromone family of *E. nobilii*, and the six pheromones E*r*-1, E*r*-2, E*r*-10, E*r*-11, E*r*-22 and E*r*-23 from the mesophilic pheromone family of *E. raikovi* (Figure 1)*.* Considering that NMR solution structures are available for all the nine proteins, and for one (*i.e.*, E*r*-1) is available also the crystallographic structure [21], we expect that these data will be of interest for in-depth studies of correlations between molecular protein structure, thermodynamic stability, and cold adaptation.

## 2. Materials and Methods

The isolation and purification of the nine proteins investigated in this paper has previously been described [22,23,24,25]. Lyophilized samples of each pheromone were dissolved in 20 mM sodium phosphate buffer at pH 6.0 and diluted to a protein concentration of 20 µM before aliquoting into a 0.1 cm path length quartz cuvette. CD experiments were recorded using the Temperature/Wavelength Scan software supplied with the Jasco 815 CD spectrophotometer. Melting curves over the range 20–95 °C were measured at a constant wavelength of 220 nm by increasing the temperature at a rate of 1.0 or 0.5 °C/min. Wavelength scans from 260–190 nm were measured at 5 °C intervals.

In additional exploratory studies, the chemical denaturants guanidine-HCl and urea were added to solutions of the mesophilic pheromones E*r*-1, E*r*-10, E*r*-22 and E*r-*23 to further test their stabilities. In particular melting curves were recorded for E*r*-1 in 7.8 M urea and 20 mM sodium phosphate at pH 6.0, 6 M guanidine-HCl and 20 mM sodium phosphate at pH 6.0, 4 M guanidine-HCl and 20 mM sodium phosphate at pH 6.0, 4.5 and 3.0, and 6 M urea at pH 3.0. Similar analyses on E*r*-10, E*r*-22 and E*r*-23 were performed using 4 M guanidine-HCl in 20 mM formic acid at pH 2.0.

## 3. Results

**Figure 2:**
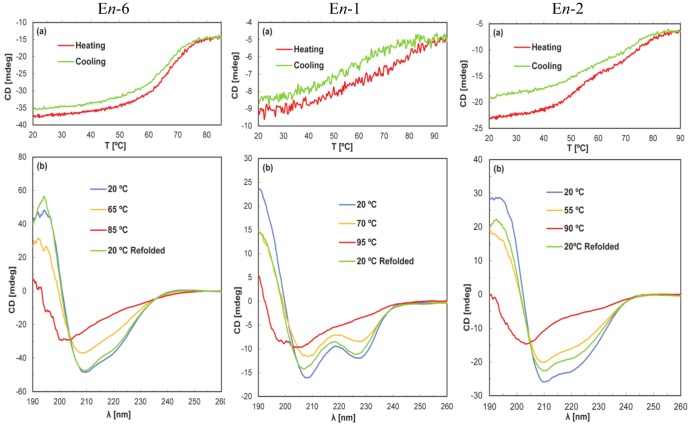
Temperature-induced denaturation of the *E. nobilii* psychrophilic pheromones E*n*-6, E*n*-1 and E*n*-2 monitored by CD spectroscopy. (**a**) Temperature variation of the signal intensity at 220 nm during heating and cooling over the range from 20 °C to 95 °C. (**b**) CD spectra at different temperatures, as indicated by the color code in the figure. The protein concentration was 20 µM in 20 mM sodium phosphate at pH 6.0, and the cell length 0.1 cm. The temperature variation of the signal intensity at 220 nm in panels (**a**) was recorded at a rate of 1.0 °C/min. Scans were recorded with a speed of 100 nm/min, in 5 °C intervals over the temperature range of panels (**a**); for improved clarity, only four traces are shown in panels (**b**).

For all the three psychrophilic pheromones E*n*-1, E*n*-2, and E*n*-6 of *E. nobilii*, the CD spectra (Figure 2) show that the regular secondary structures are unfolded at 95 °C, and that this unfolding is reversible upon cooling of the solutions to the starting temperature at 20 °C. Nevertheless, among the individual proteins there are appreciable variations with regard to the shape of the thermal unfolding curves. For E*n*-6 a nearly symmetrical sigmoidal denaturation curve was observed with a midpoint near 65 °C, and a sigmoidal curve was also obtained for the refolding upon cooling of the solution; in addition, only a very small loss of protein was recorded during the unfolding/refolding procedure. On the other hand, E*n*-1 and E*n*-2 showed more sluggish unfolding transitions, and for E*n*-2 there was an indication that the unfolding and refolding processes involve equilibria between more than two states. For the present qualitative survey of the stability of these three proteins we retain that, similar to E*n*-6, the regular secondary structures in E*n*-1 and E*n*-2 are unfolded at 95 °C and refolded upon cooling of the solutions to 20 °C.

**Figure 3:**
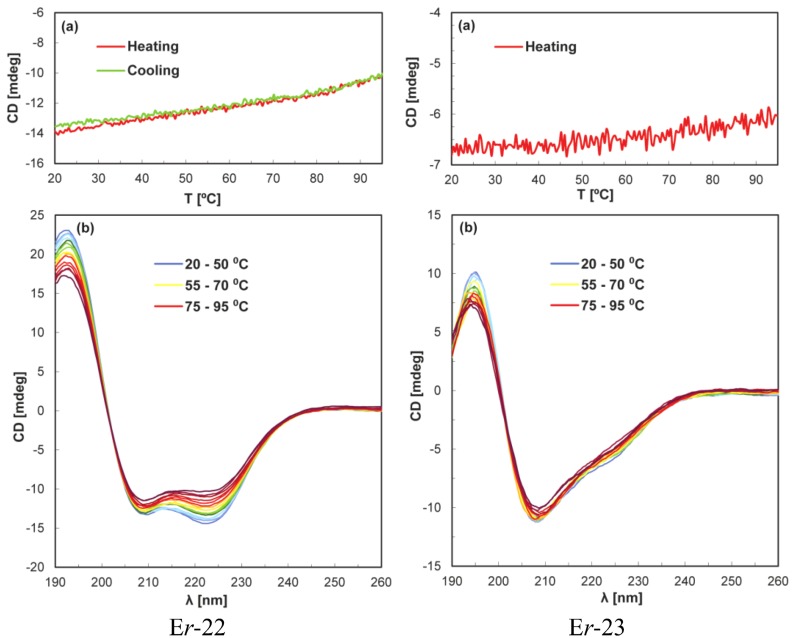
Temperature-induced denaturation of the *E. raikovi* mesophilic pheromones E*r*-22 and E*r*-23 monitored by CD spectroscopy. E*r*-22 has been taken as representative of the other *E. raikovi* pheromones E*r*-1, E*r*-2, E*r*-10 and E*r*-11. Same experimental conditions and presentations as in Figure 2, except that all the spectra recorded in 5 °C temperature intervals are shown in panels (**b**).

In the family of the six mesophilic pheromones E*r*-1, E*r*-2, E*r*-10, E*r*-11, E*r*-22, and E*r*-23 from *E. raikovi*, the regular secondary structures manifested in the CD spectra at 20 °C were found to be maintained up to the highest temperature studied (Figure 2). The temperature dependence of the signal intensity at 220 nm did not provide evidence for unfolding, and for E*r*-22 the small reduction of the signal intensity at the higher temperatures appeared to be fully reversible upon solution cooling. The data recorded for this pheromone are representative of the observations made with the other pheromones E*r*-1, E*r*-2, E*r*-10, and E*r*-11. Also the pheromone E*r*-23 showed qualitatively similar behavior, but it was clearly the most stable member of the *E. raikovi* mesophilic protein family since it did not undergo denaturation (Figure 3).

In view of the high thermal stability of the *E. raikovi* pheromones in neutral aqueous solution, we also performed exploratory experiments with the addition of chemical denaturants (see Materials and Methods). These experiments provided further indications of the remarkably high stability of these proteins. We did not observe their full unfolding in the temperature range 20–95 °C with the solution conditions listed in the Materials and Methods section, although partial melting within this temperature range was observed for some of them.

## 4. Discussion

The psychrophilic and mesophilic pheromone families of the protozoan ciliate *Euplotes* studied here are both represented by single-domain small disulfide-rich proteins, which have usually been shown to be outstandingly stable with regard to thermal denaturation in aqueous solution [26,27,28]. However, despite their extensive homology on the level of the amino acid sequences and three-dimensional structures, the psychrophilic *E. nobilii* pheromones showed a significantly lower thermal stability than their mesophilic *E. raikovi* counterparts. This finding coincides with observations derived from comparisons between other psychrophilic and mesophilic homologous proteins [29,30,31,32,33]. However, while these comparisons are essentially based on individual proteins from distantly related organisms, our finding involves families of proteins with known NMR solution structures [11,12,13,14,15,16,17,18,19,20,21] from two closely related species [34,35]. It therefore provides data which, at least in principle, are more reliable (being unaffected by the evolutionary noise which is intrinsic to comparisons between distantly related systems) for further detailed analyses by other groups of researchers who are interested in studying the correlations between protein structure, thermodynamic stability, and cold-adaptation.

As previously discussed [3,10,21], the two homologous protein families of *E. nobilii* psychrophilic pheromones and *E. raikovi* mesophilic pheromones differ significantly in the composition of polar, hydrophobic and aromatic amino acids, in global hydrophilicity and hydrophobicity, as well as in various aspects of their three-dimensional structures. It will now be interesting to explore the extent to which these physico-chemical and structural variations can be related to variations of the stability of the folded proteins. Two structural features distinctive of the two pheromone families appear to be particularly promising starting points for further investigations into the structural basis of the observed differences in the thermal stability of these proteins. One is the *N*-terminal elongation of 10 to 12 residues, which is common to all the *E. nobilii* pheromones and has no counterparts in any of the *E. raikovi* pheromones. It includes only a 3_10_-helical turn as regular secondary structure and is devoid of connections through disulfide bonds to other parts of the proteins. Secondly, there is a higher density of disulfide bonds in the mesophilic *E. raikovi* pheromones than in the psychrophilic ones of *E. nobilii*. On average, the *E. raikovi* pheromones contain one disulfide bond per 13 amino acid residues, while in the *E. nobilii* pheromones there is one disulfide bond per 15 residues. In particular, the high density of one disulfide bond per 10 residues appears to be the dominant feature that makes E*r*-23 most stable among the *E. raikovi* pheromones, notwithstanding that E*r*-23 mimics the *E. nobilii* psychrophilic pheromones with regard to the contents of polar and hydrophobic residues as well as the aliphatic index.

## 5. Conclusions

The key observation reported in this paper is that the *E. nobilii* and *E. raikovi* pheromone families show a thermal denaturation behavior that is uniform among their members and significantly divergent between them. The mesophilic *E. raikovi* pheromones have higher stability by at least 30 °C when compared to the psychrophilic *E. nobilii* pheromones. Given the small size of these water-borne signaling proteins and the availability of their three-dimensional structures, the data presented in this communication should be of interest for systematic investigations of protein adaptation to cold environments.
